# Norovirus gastroenteritis general outbreak associated with raw shellfish consumption in South Italy

**DOI:** 10.1186/1471-2334-4-37

**Published:** 2004-09-21

**Authors:** Rosa Prato, Pier Luigi Lopalco, Maria Chironna, Giovanna Barbuti, Cinzia Germinario, Michele Quarto

**Affiliations:** 1 Dipartimento di Scienze Mediche e del Lavoro, Section of Hygiene, University of Foggia, Italy; 2 Dipartimento di Medicina Interna e Medicina Pubblica, Section of Hygiene, University of Bari, Italy; 3 Dipartimento di Scienze Biomediche ed Oncologia Umana, Section of Pathology, University of Bari, Italy

## Abstract

**Background:**

Despite *Noroviruses *(NV, previously "Norwalk-like viruses") being a leading cause of acute gastroenteritis outbreaks, the impact of NV infection is at present unknown and little information is available about strains circulating in Italy. In April 2002 an outbreak of gastroenteritis occurred in the province of Bari (South-east Italy), involving several households.

**Methods:**

A retrospective cohort study was performed in order to assess risk factors associated with illness. All households where a case occurred were included in the study. Faecal specimens were collected from ill individuals. NV-specific RT-PCR was performed. Eleven samples of mussels were collected from fish-markets involved in the outbreak. A nested PCR was used for mussel samples.

**Results:**

One hundred and three cases, detected by means of active surveillance, met the case definition. Raw shellfish eating was the principal risk factor for the disease, as indicated by the analytic issues (Risk Ratio: 1.50; IC 95%: 1.18 – 1.89; p < 0.001). NVs were found by means of RT-PCR of all the stool specimens from the 24 patients tested. Eleven samples of shellfish from local markets were tested for the presence or NVs; six were positive by nested PCR and genotypes were related to that found in patients' stools.

**Conclusion:**

This is the first community outbreak caused by NVs related to sea-food consumption described in Italy. The study confirms that the present standards for human faecal contamination do not seem to be a reliable indicator of viral contaminants in mussels.

## Background

*Norovirus *(NV, previously "Norwalk-like viruses"), one of four genera in the *Caliciviridae *family, includes a group of morphologically similar but genetically different single-stranded RNA viruses. NVs represent the most important cause of non-bacterial gastroenteritis worldwide. In industrialised countries NVs may be responsible for up to 80% of all outbreaks of gastroenteritis [1]. Outbreaks may affect all age groups and generally occur in crowded communities such as restaurants, tourist resorts, hospitals, schools and nursing homes.

Contaminated food or water commonly represents the main source of infection. Epidemics spread by the faecal-oral route, even if transmission may also occur through direct person-to-person contact or aerosolised viral particles.

The incubation period of NV gastroenteritis is 24–48 hours and symptoms include vomiting, diarrhoea, abdominal pain, low-grade fever, headache and myalgia.

Italy has no specific surveillance system for viral gastroenteritis and laboratory diagnosis is only carried out in a few cases. Therefore the impact of NV infection is currently unknown and little information is available about circulating strains. Outbreak investigations are usually performed by local health units. Gastroenteritis notifications are often too delayed to identify the etiologic agent and source of infection correctly.

Puglia, a region in the South-East of Italy, has about four million inhabitants. Bari (about 1,500,000 people in the province) is the capital. Raw mussels are largely consumed in Bari and, in general, in Puglia, especially at Christmas and Easter. In 1994 raw seafood consumption was the source of a small cholera outbreak. Moreover, hepatitis A is endemic in this region.

This report describes a large outbreak of NV gastroenteritis that involved several households in Bari province.

## Methods

### Descriptive and analytical studies

The outbreak occurred during the Easter holidays, between March 31^st ^and April 7^th ^2002, in the province of Bari (Puglia region, South Italy). In Italy, at Easter many people eat in restaurants and the day after Easter most people go picnicking. In the province of Bari seafood (especially mussels) are often consumed on these occasions. The Regional Epidemiological Office, alerted by the Local Health Unit, performed a field study, collecting data from all General Practitioners and all Emergency Units in the province. Active surveillance was conducted until April 15^th ^2002 (one week after the last case onset). The aim of the investigation was to describe the outbreak and to identify the etiologic agent, the source of infection and the means of transmission.

A case was defined as illness in a resident in the area during the period March 31^st ^– April 7^th^, who had diarrhoea (three or more loose stools in any 24 hour period) or vomiting (at least one episode). Fever greater than or equal to 38°C, abdominal pain or nausea were considered additional symptoms. A "probable" secondary case was defined as illness in a household with onset of symptoms more than 24 hours after the primary case.

A retrospective cohort study was performed in order to assess risk factors associated with illness.

All households where a case occurred were included in the study.

Information, collected by means of a standardised questionnaire, included: 1) demographic individual characteristics such as age, gender, occupation; 2) type of food consumed during the last 72 hours before onset of symptoms; 3) if ill, type, date and time of onset of symptoms.

To assess the association between food consumption and disease, relative risks (RR) and 95% confidence intervals (95% CI) were calculated. Age and gender were compared between ill and unaffected individuals by the chi squared and Mann-Whitney tests. A p-value less than 0.05 was considered significant. Variables associated to the illness in the univariate analysis were included in the stratified analysis; summary RR and 95% CI were calculated from the formulas of Greenland and Robins [2]. Collected data were analysed by using Epi Info 6.04 (Centres for Disease Control and Prevention, Atlanta, GA) and Statview 4.0 (Sas Institute Inc., Cary, NC) software.

### Laboratory investigations

Faecal specimens were collected from ill individuals. Part of the specimens was refrigerated and processed within 12 hours to detect ova and parasites by direct microscopy and common bacteria by standard methods. The rest was stored at -20°C until examination by NV-specific reverse transcription-polymerase chain reaction (RT-PCR).

Viral nucleic acid extraction and purification from stool specimens was performed as previously reported [3]. RT-PCR was carried out with the primers JV12-SM31 specific for the polymerase gene of NV [4].

Eleven samples of mussels were collected from two fish-markets from which the cases had bought the shellfish they consumed. Mussel samples were also processed within 12 hours to detect common bacteria by standard methods.

Then a nested PCR was used. The procedure for mussel processing as well as viral RNA extraction and purification has been previously described in full [5].

The primers used for first round PCR were JV12 and SM31. Nested PCR was carried out with the use of the primers SR33 for negative strand DNA synthesis and SR48 and SR46 for positive strand synthesis of genogroup I (GI) and genogroup II (GII) sequences, respectively [6].

The 333-bp or 123-bp amplification products from cases and from mussels were subjected to sequencing with PCR primers. When required, cloning was carried out on PCR products. Sequences obtained were aligned with those available in the GenBank.

## Results

### Epidemiological issues

One hundred and three subjects met the case definition; 22 of them were defined as "probable" secondary cases. Fifty eight (56.3%) of the 103 cases were female; the mean age was 42.6 years (table 1).

The clinical pattern of the disease was characterised by the presence of vomiting (84.5%), nausea (58.3%), diarrhoea (53.4%), abdominal pain (47.6%), fever (16.5%). No difference was observed in the clinical pattern by gender. Age was significantly higher in ill individuals with severe diarrhoea (lasting more than 24 hours or undergoing specific treatment) (46.3 vs 38.3 years; p = 0.036). No difference in age was found for the other symptoms.

**Figure 1:**
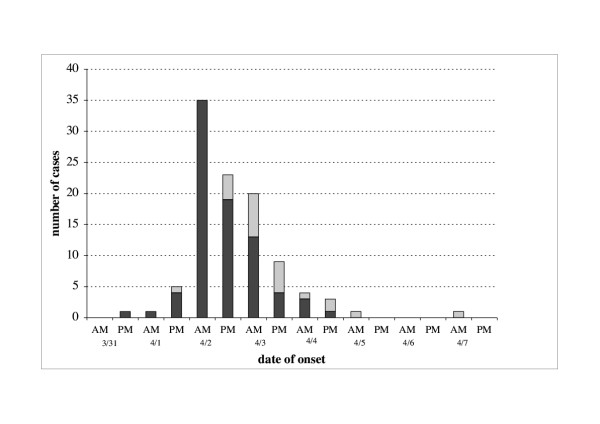
Distribution of cases of NV gastroenteritis (n= 103) by onset time, March-April 2002. Probable secondary cases are coloured in grey.

The outbreak started on March 31^st ^at 5,00 pm and lasted eight days. The epidemic curve shows a single peak on April 2^nd ^(35 cases between 1:00 and 12:00 AM) and a right tail probably due to secondary cases (figure 1). Incubation time was not calculated because it was not possible to state a single exposure time. In fact this outbreak could represent the result of multiple small household outbreaks, the source of which could have been either a restaurant meal (on Easter Sunday) or a picnic the day after Easter.

All cases belonged to 30 households that included 139 individuals in all. All members of each household ate together at least once (at the same restaurant or during the same picnic) in the days before the onset of symptoms of relatives. All 36 healthy subjects were interviewed. Nineteen (52.8%) of them were female, the mean age was 42.5 years. Gender and age were not significantly different between healthy and ill individuals (table 1).

An attack rate of 74.1% (103/139) was observed. In the univariate analysis, the association between raw mussel consumption and illness was significant (RR: 1.50; 95% CI: 1.18 – 1.89; p < 0.001). The attack rate according to raw mussel consumption was 86.3% (69/80).

Even cooked mussel consumption was associated with illness in the univariate analysis (RR: 1.53; 95% CI: 1.05–2.23; p = 0.003). No other food was associated with illness (table 2).

Stratified analysis showed that cooked mussel consumption was significantly associated with illness only among those who did not eat raw seafood (RR: 3.04; 95% CI: 1.26 – 7.30; p < 0.001). Such association was not significant in the "ate raw mussels" stratum (RR: 0.84; 95% CI: 0.76 – 0.93; p > 0.05). The relative risk according to cooked mussel consumption was 1.38 (95% CI: 1.00 – 1.91) after correction for raw mussels consumption (table 3).

Subjects defined "probable secondary cases" were considered ill according to the risk assessment. In fact, we could not be certain that "probable secondary cases" were actually co-primary cases; on the other hand, the association between raw mussels and illness was stronger excluding from analysis such cases (RR: 1.63; 95% CI: 1.22–2.18; p < 0.001).

### Laboratory issues

Both stool and mussel samples were negative for parasites and bacterial enteropathogens. All stool samples from 24 cases were positive for NV by RT-PCR and 5 of 11 mussel samples by nested PCR. The sequences of strains revealed great heterogeneity. In fact, the simultaneous occurrence of GI and GII viruses within the same outbreak was observed. One sample of mussels showed the presence of a mixed genogroup. Sequence analysis showed that strains from 19 cases and 3 mussel samples had identical sequences and belonged to GII, clustering narrowly to the strain NLV/Tarrag/238/2001/Sp, thus strengthening the epidemiologic link of the mussels to the cases. The nucleotide sequences from three cases and 2 mussel samples formed two other distinct clusters showing the best fit with the strains Saitama U25 and Khs1-1997-JP. Sequences from two further cases and one mussel sample were assigned to genogroup I and showed a high degree of identity with the strain NLV/Steinbach/EG/2001/CA.

Three familial groups consisting of 7 cases and three members of a further family group from the outbreak showed identical sequences and were classified as NV GII (the main cluster of 19 cases). A fourth member of the latter family group was positive for one of the two GI strains. The other strains investigated by sequence analysis were unrelated to those from these familial groups.

## Discussion

In Italy a national NV outbreaks database does not exist, although NVs are the leading cause of gastroenteritis outbreaks in Europe as in the rest of the western countries [7]. Moreover, to our knowledge, this is the first NV community outbreak to be confirmed in Italy [8]. The actual number of NV gastroenteritis cases is underestimated since the illness is often mild and diagnostics are provided only in a few laboratories. Despite these factors, the knowledge of circulating strains and the recognition of the common vehicles of infection are of primary importance for prevention of such disease.

This investigation showed the causative role of mussel consumption in the outbreak, confirmed by laboratory tests on both stools and food samples.

The heterogeneity of viral strains associated with the consumption of contaminated mussels is no surprise due to the peculiar features of shellfish. In fact, bivalve shellfish are filter feeders and tend to accumulate whatever pollutants are in the water which can result in viral contamination from a multitude of possible sources affecting many individuals. In any case, further studies should be carried out to clarify this issue [9]. The low relative risks we found could be due to the study design. In fact, the selection of households where a case occurred could underestimate the association. However this study design became necessary because of the unfeasibility of an open cohort study.

In the 1990s, NVs were identified as the primary pathogen associated with shellfish-borne gastroenteritis in the United States [10]. Since then many studies have confirmed the role of shellfish in the spread of NV infection.

Raw shellfish consumption is very frequent among the people of Puglia. Mussels sold in Puglia come from a large number of suppliers: from countries within the European Union (EU) and from other Mediterranean countries. The frequent shellfish consumption had already been blamed for the cases of cholera that occurred in Puglia in 1994, during the Christmas period, when a major epidemic was reported in Albania, on the opposite coast of the Adriatic sea [11]; moreover, mussels have been identified as the principal vehicle during recurring hepatitis A epidemics [5,12,13]. A significant presence of HAV in mussels sold in Puglia has recently been demonstrated by experimental evidence [3,5], even in samples negative for standard microbiological controls. In Puglia informative campaigns and routine microbiological controls on seafood are currently performed. The effectiveness of these interventions seems to be scarce.

Moreover, our data showed cooked mussels also played a significant role. In fact cooking might not completely inactivate NVs and because of the low infectious dose, even a limited residual contamination can result in illness [14].

The epidemic curve shows a usual pattern consistent with a common point source. However, person to person transmission may have played a role in the last phase of the outbreak. On the other hand, such a means of transmission of NV is well documented.

This investigation confirms the importance of field study in gastroenteritis outbreaks, as well as obtaining stool specimens from patients and food samples for laboratory analysis in a short time [15].

The present standards for human faecal contamination do not seem to be a reliable indicator of viral contaminants in mussels [5]. Seafood samples analysed during this investigation were all negative for the presence of common bacteria. On the other hand, to protect consumers it would be necessary to use a molecular index of the human contamination. In such cases reference laboratories with high-technology facilities would be required. The lack of such laboratories could be an obstacle to implementing a routine control system based on molecular tests.

## Conclusions

This episode confirms that large NV outbreaks occur in Italy but only an accurate investigation can recognise this pathogen and that current regulations and commercial practices need to be revised to assure the safety of shellfish consumption and to improve control of future outbreaks.

## Competing interests

None declared.

## Authors' contributions

MC and GB carried out microbiological assays and drafted the manuscript. CG participated in the design of the study and performed the statistical analysis. MQ conceived the study as well as participating in its design and coordination. All authors have read and approved the final manuscript.

## Pre-publication history

The pre-publication history for this paper can be accessed here:



## Figures and Tables

**Table 1:** Study population by age and gender.

	Ill	Not Ill	p
Age (mean ± standard deviation)	42.6 ± 20.1	42.5 ± 20,8	>0.05*
Age range	5 – 80	6 – 85	
Gender (M/F)	45/58	17/19	>0.05**
Total	103	36	

* unpaired t

test ** chi square

**Table 2:** Attack rates, relative risk, confidence intervals and chi-squared p value according to exposure.

Exposure	No. Exposed	No. Cases (n = 103)	Attack Rate (%)	Relative Risk	95% Confidence Interval		p value
raw mussels	80	69	86.3	1.50	1.18	1.89	<0.001
cooked mussels	112	89	79.5	1.53	1.05	2.23	0.003
eggs	16	13	81.3	1.10	0.85	1.43	>0.05
oven cooked pasta	62	43	69.4	0.89	0.73	1.09	>0.05
grilled sausage	51	36	70.6	0.93	0.75	1.15	>0.05
grilled meat	89	65	73.0	0.96	0.79	1.17	>0.05
grilled lamb	70	52	74.3	1.01	0.83	1.22	>0.05
dairy milk products	77	54	70.1	0.89	0.73	1.08	>0.05
cheese	49	35	71.4	0.93	0.76	1.15	>0.05
fresh vegetables	85	65	76.5	1.09	0.88	1.34	>0.05
pastries	53	40	75.5	1.03	0.84	1.26	>0.05

**Table 3:** Relative risk and 95% confidence interval according to mussels consumption: stratified analysis.

	No. Exposed	No. Cases	Attack Rate (%)	Relative Risk	95% Confidence Interval		p value
*eating raw mussels = "yes" (n = 80) *eating cooked mussels	70	59	84.3	0.84	0.76	0.93	>0.05
*eating raw mussels = "no" (n = 59) *eating cooked mussels	42	30	71.4	3.04	1.26	7.30	<0.001
Summary Risk Ratio				1.38	1.00	1.91	
